# Locomotor preferences in terrestrial vertebrates: An online crowdsourcing approach to data collection

**DOI:** 10.1038/srep28825

**Published:** 2016-07-06

**Authors:** John Lees, James Gardiner, James Usherwood, Robert Nudds

**Affiliations:** 1 Faculty of Life Sciences, University of Manchester, Manchester, M13 9PT, UK; 2 School of Computing, Science and Engineering, University of Salford, Salford, M5 4WT, UK; 3 Structure and Motion Laboratory, The Royal Veterinary College, University of London, North Mymms, Hatfield, Herts, AL9 7TA, UK

## Abstract

Understanding how animals move within their environment is a burgeoning field of research. Despite this, relatively basic data, such as the locomotor speeds that animals choose to walk at in the wild, are sparse. If animals choose to walk with dynamic similarity, they will move at equal dimensionless speeds, represented by Froude number (Fr). Fr may be interpreted from simple limb kinematics obtained from video data. Here, using Internet videos, limb kinematics were measured in 112 bird and mammal species weighing between 0.61 and 5400 kg. This novel method of data collection enabled the determination of kinematics for animals walking at their self-selected speeds without the need for exhaustive fieldwork. At larger sizes, both birds and mammals prefer to walk at slower relative speeds and relative stride frequencies, as preferred Fr decreased in larger species, indicating that Fr may not be a good predictor of preferred locomotor speeds. This may result from the observation that the minimum cost of transport is approached at lower Fr in larger species. Birds walk with higher duty factors, lower stride frequencies and longer stance times compared to mammals at self-selected speeds. The trend towards lower preferred Fr is also apparent in extinct vertebrate species.

Terrestrial locomotion forms a significant portion of daily activity for many vertebrates and is energetically costly, yet studies of animals walking in the wild at self-selected speeds are few^1^,^2^,^3^. Walking animals may be compared based upon Froude number (Fr = *u*^2^/*gh*, where *u* is velocity, *g* is the acceleration due to gravity and *h* is functional hip height), a dimensionless parameter which allows comparisons to be made among species, taking regard of their size, by standardising the ratio of centripetal to gravitational forces^4^. If animals are geometrically similar and moving at equal Fr, then they are moving in dynamically similar ways. It follows that if there is an optimal pattern of movement that minimizes the gross metabolic cost of transport (CoT), we might expect animals to move at equal values of Fr. In practice, perfect geometric similarity is not found across species. However, many of the predictions of the dynamic similarity hypothesis are supported for cursorial mammals (most mammals of mass greater than 5 kg)^4^,^5^. For example, gait transitions occur at similar values of Fr^4^. Unfortunately, there are few comprehensive kinematic studies that include a diversity of species for which hip height and body mass (*M*_b_) data are reported. Those that do include such data demonstrate that kinematic parameters change predictably with Fr across species of different size and therefore they may be used to infer Fr. In mammals and birds, larger species were shown to walk with lower duty factors (DF, the proportion of the stride during which the foot is on the ground) at equal values of Fr^6^,^7^ (proportional to *M*_b_^−0.023 to −0.025^ Fig. 1a) and one study in birds with *M*_b_ ranging from 0.045–90 kg demonstrated that at the same Fr, larger animals walk with lower stride frequencies *f*_stride_ (proportional to *M*_b_^−0.12 to −0.13^, Fig. 1b) higher stance durations (*t*_stance_, s, proportional to *M*_b_^0.05 to 0.1^, Fig. 1c) and higher swing durations (*t*_swing_, s, proportional to *M*_b_^0.16 to 0.18^, Fig. 1d)^7^. It is important to note that scaling exponents are presented as a range as the relationship between kinematic parameters and Fr differs slightly depending on the value of Fr. An equivalent dataset for mammals is unavailable although, interestingly, *f*_stride_ is proportional to *M*_b_^−0.14^ in mammalian species moving at the trot-gallop transition speed, an exponent similar to that of birds when walking at the same Fr. Despite the centrality of the dynamic similarity hypothesis to our understanding of terrestrial locomotion, it is unclear if the preferred walking speeds and associated kinematics of wild animals are coincident with similar Fr.

Kinematic parameters are intimately linked to the work requirements of walking and therefore to the CoT (assuming a relationship between mechanical and metabolic cost) but are rarely quantified outside of the laboratory. Such field data, in which animals are self-selecting their walking speeds, are critical in understanding the pattern of energy use of animals in their natural environments. Contra to the predicted kinematics when animals are walking at dynamically similar speeds (Fr), self-selected DF increases with size in mammals with a slope proportional to *M*_b_^0.02^ (Fig. 1a). Equivalent data for birds are unavailable. This increase in DF appears to result from larger species taking relatively longer stances as opposed to shorter swing durations (Fig. 1c,d). *f*_stride_ scales as approximately *M*_b_^−0.16^ in freely walking mammals^1^,^3^,^8^ and as *M*_b_^−0.4^ in birds^2^ (Fig. 1b), a slope lower than that predicted if animals were walking at the same Fr^7^. The latter exponent, however, may be influenced by low sample sizes of species with diverse morphologies^9^ and a low number of steps analysed (only 14 of the 25 species investigated had more than 5 steps analysed)^2^. Together, the available kinematic data suggest that animals may not self-select speeds that are dynamically similar, however these data are sparse.

To date, a comprehensive study of the self-selected walking speeds of terrestrial species and the associated kinematics is lacking. Here, using user-generated content from an online video database, the walking kinematics of a range of birds and mammals was quantified to test the hypothesis that at self-selected walking speeds, animals move in dynamically similar ways. Additionally, because the idea of dynamic similarity is underpinned by geometric similarity, the hypothesis that dynamic similarity would only hold within class groups was also investigated.

## Results

DF increased at a faster rate with *M*_b_ in birds (proportional to *M*_b_^0.03^) than in mammals (proportional to *M*_b_^0.02^) and was higher in birds at any given *M*_b_ (Fig. 2a, Table 1 and Supplementary Table S2). In contrast, the incremental change in self-selected *f*_stride_ with *M*_b_ was similar for birds and mammals (Fig. 2b), decreasing as *M*_b_^−0.15^ in both class groups, but was 0.4 Hz (95% CI = [0.19, 0.39]) lower in birds at all *M*_b_. Again, the incremental change in *t*_stance_ with *M*_b_ was similar in both birds and mammals (Fig. 2c) increasing as *M*_b_^0.17^ in both groups. Birds, however, walked with a longer *t*_stance_ than mammals (mean difference = 0.18 s, 95% CI = [−0.5, −0.29]). *t*_swing_ was similar in birds and mammals and scaled as *M*_b_^0.11^ in both (Fig. 2d).

## Discussion

**Figure 1:**
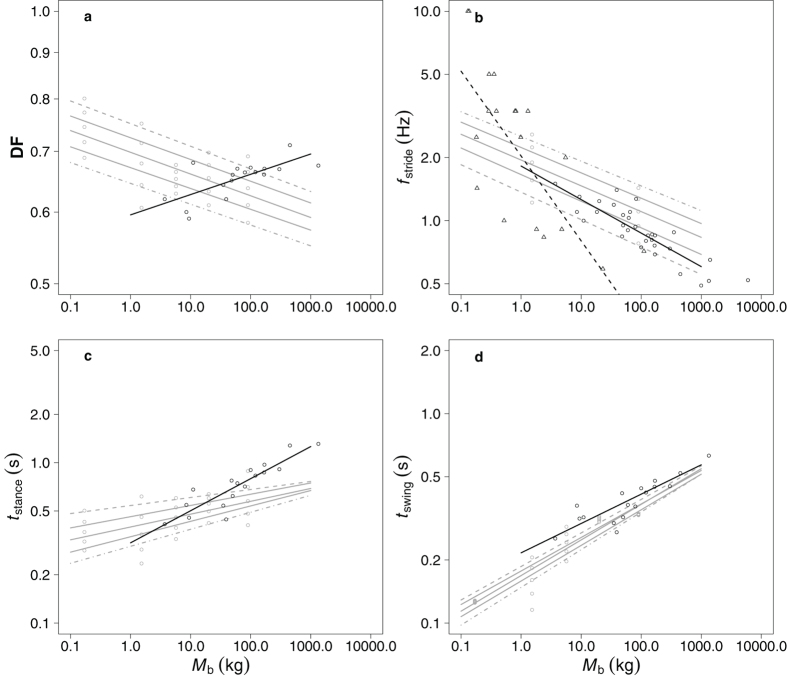
Predicting the relationship between gait kinematics and Froude number. Log-log plots of (**a**) duty factor (DF), (**b**) stride frequency (*f*_stride_, Hz) (**c**) stance duration (*t*_stance_, s) and (**d**) swing duration (*t*_swing_, s) plotted against body mass (*M*_b_) taken from the literature. Kinematic data are shown for a variety of birds walking on treadmills^7^ at different Froude numbers (grey circles, grey lines) from 0.1 (dashed lines) to 0.5 (dotted and dashed lines) in 0.1 increments. Existing data for mammals^1^,^3^,^8^ (black circles, black solid lines) and birds^2^ (black triangles, black dashed line) walking at self-selected speeds are shown for comparison.

**Figure 2:**
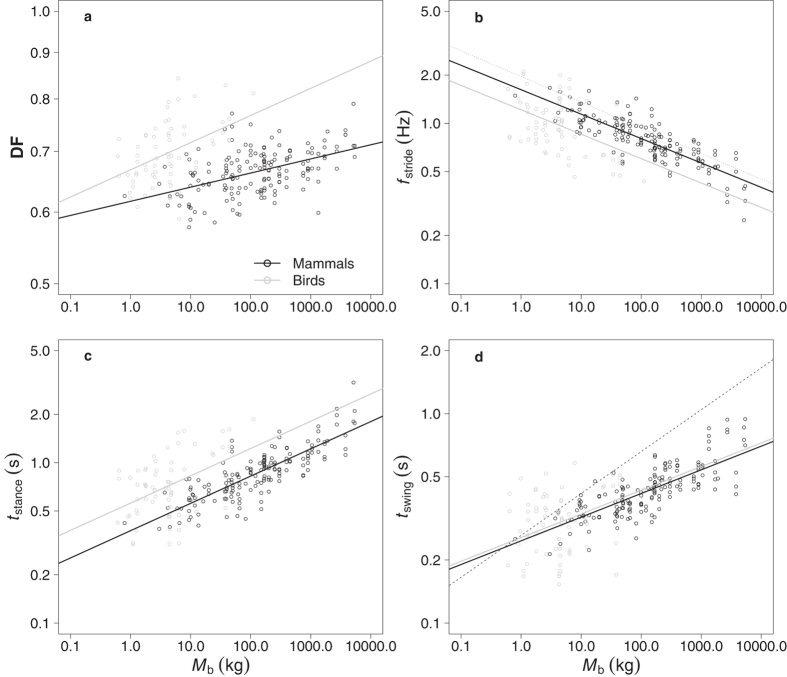
Preferred gait kinematics in terrestrial vertebrates. Log-log plots of (**a**) duty factor (DF), (**b**) stride frequency (*f*_stride_, Hz), (**c**) stance duration (*t*_stance_, s) and (**d**) swing duration (*t*_swing_, s) against body mass (*M*_b_, kg). The dotted line in (**b**) represents the line of best fit through the only comparable data for African mammals^3^. The dashed line in (**d**) represents the natural pendular period for mammalian limbs^21^. The lines fitted through the data are derived from the ANCOVA output and are: DF = 0.62 *M*_b_^0.02^ (mammals), DF = 0.67 *M*_b_^0.03^ (birds); *f*_stride_ = 1.62 *M*_b_^−0.15^ (mammals), *f*_stride_ = 1.21 *M*_b_^−0.15^ (birds); *t*_stance_ = 0.38 *M*_b_^0.17^ (mammals), *t*_stance_ = 0.56 *M*_b_^0.17^ (birds); *t*_swing_ = 0.25 *M*_b_^0.11^ (mammals), *t*_swing_ = 0.26 *M*_b_^0.11^ (birds). More details of the analyses can be found in Supplementary Table S2.

The present study is the first to quantify locomotor kinematics at self-selected speeds across such a diversity of species, particularly birds. We reject the hypothesis that animals prefer to walk at dynamically similar speeds as defined by Fr. Previous data indicate that large animals have a lower DF than small animals walking at the same Fr (Fig. 1a)^4^,^6^. Here, preferred DF increases with *M*_b_ in both mammals and birds (Fig. 2a), providing strong evidence that larger animals walking relatively more slowly than small species.

**Figure 3:**
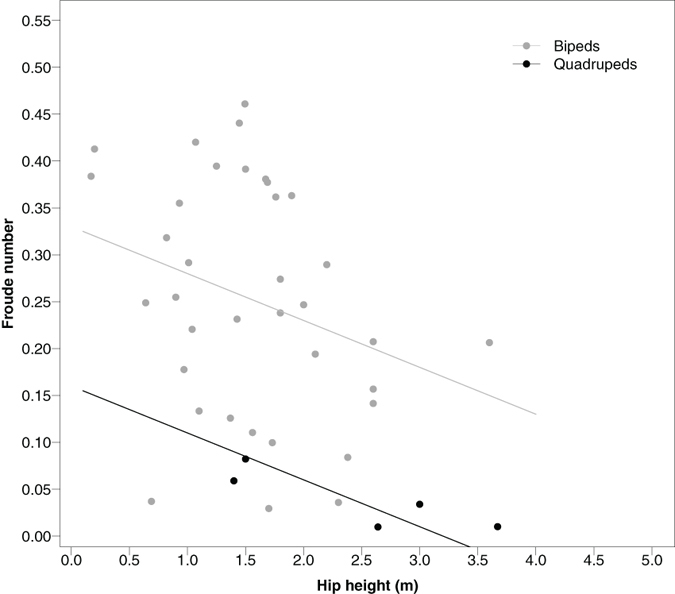
Estimated Froude numbers of bipedal and quadrupedal dinosaurs. Froude numbers estimated from dinosaur trackways over a large size range of quadrupeds and bipeds mirror our findings that smaller vertebrate species walk relatively more quickly (i.e. with a higher Froude number) than larger species. The references for these data can be found in Supplementary Table S3.

A preference for lower Fr with a coincident increase in DF has been demonstrated in 9 species of cat and the present data suggest that this pattern may be observed broadly across terrestrial vertebrates. Preferred *f*_stride_ decreased with *M*_b_^−0.15^, an exponent lower than the lowest value for birds when moving at an equal Fr of 0.1 (Fig. 1b, *f*_stride_ ~ *M*_b_^−0.13^) again suggesting that larger species walk at relatively slower speeds. This exponent is not as low as that found previously in birds^2^ or mammals^1^,^3^,^8^. These studies, however, contain much smaller and less diverse datasets compared to the data presented here. The relatively higher DF and relatively lower *f*_stride_ in larger species indicating a lower Fr appears to be due to a combination of both relatively longer *t*_stance_ and relatively shorter *t*_swing_ in large animals. This is contra to the previous data in mammals that suggested a role of *t*_stance_ alone and may result from differences in the passive swing properties of the legs with increasing size as well as differences in the active propulsion of the limbs. Regardless of the underlying mechanisms, the preference for relatively slower speeds at increased size appears to be a central feature of locomotion in terrestrial vertebrates despite large variations in morphology and life histories. This preference is even observed in extinct bipedal and quadrupedal dinosaurs when their preferred Froude is estimated from fossilized trackways (Fig. 3, Supplementary Table S3).

**Figure 4:**
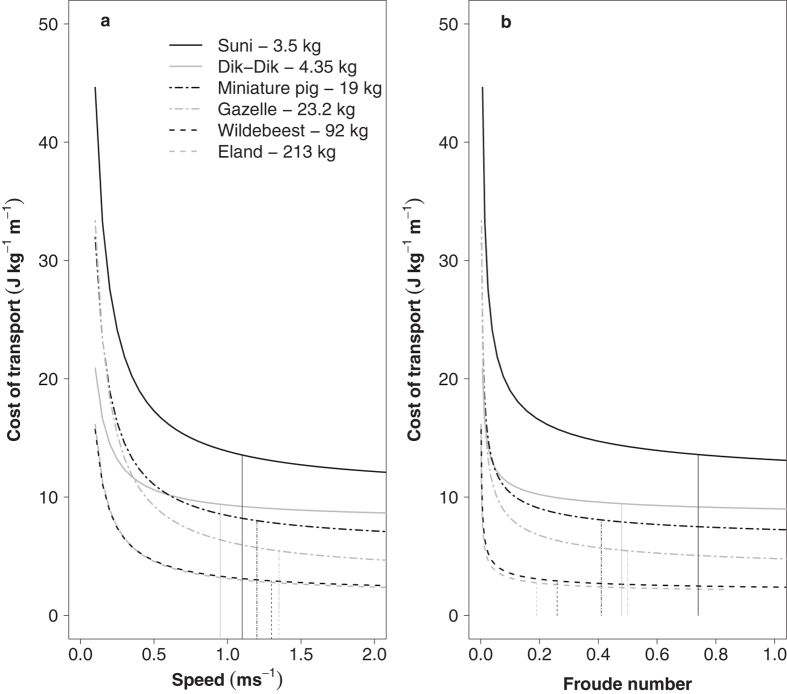
The energetics of moving at different Froude numbers shown by modelling gross cost of transport (J kg^−1^ m^−1^) as (**a**) a function of absolute speed (ms^−1^) and (**b**) Froude number, across a size range of quadrupedal mammals (energetic data are taken from^10^ and hip height data are taken from^12^). Vertical lines indicate the speed/Froude values beyond which cost of transport values change by less than 1%. The observation that larger animals approach their minimum cost of transport at lower Froude numbers may explain why they walk relatively more slowly than smaller species.

In general, the rate of oxygen consumption (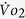, ml O_2_ s^−1^ kg^−1^) increases linearly with walking speed and the rate of this increase (the CoT_min_) is negatively correlated with *M*_b_^10^. 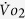 may be used to determine the CoT for species of different size (Fig. 4), by conversion to metabolic power (assuming an energetic equivalent of 20.1 J per ml of O_2_^10^). When plotted against speed, the asymptotes of the calculated curves occur at higher absolute speeds with increasing *M*_b_, although this pattern is weak (Fig. 4a). There is, however, a decrease in the Fr at which the CoT_min_ for walking is approached in animals at larger sizes (Fig. 4b). This downward shift in optimal Fr can be observed in humans walking with artificially elongated limbs^11^ and may result from the relatively longer effective limb lengths of large species for a given weight^12^,^13^. This downward shifting energetically optimal Fr may, in part, account for the lower relative speed preferences of larger birds and mammals. It remains unclear, however, why animals don’t walk at their maximum walking speed at which DF approaches 0.5 and CoT is at its theoretical minimum. Either the CoT is not minimal at the fastest walking speeds, as is the case in larger species of bird^14^ and mammal^15^ which show U-shaped CoT curves, or there are non-energetic components of speed preference. For example, animals may have a size dependent optimal intrinsic frequency above and below which locomotor reflex responses are compromised^16^. In small species, behavioral adaptations towards reducing exposure to predation and the time travelling between foraging sites (i.e. moving relatively quickly) may be additional non-energetic drivers of higher preferred walking speeds. Alternatively, larger species may walk relatively more slowly in order to minimise the chances of suboptimal foot placement, decreasing the chance of falling, which may be more costly to larger species.

Although many locomotor studies focus on stance phase mechanics, inertial forces on the limb during the swing phase of walking may also influence preferred locomotor speeds^17^. Given the cost associated with swinging the limbs^18^,^19^, minimizing this source of work may contribute to self-selected locomotor speeds and kinematics in walking species. In order to mitigate the cost of swinging the limbs, animals may move with swing durations matching or proportional to the natural pendular periods (NPP) of their limbs^3^. This passive swing is predicted by compass gait mechanics and is seen, for example in waddling birds^20^. The preferred mammalian swing durations reported here are significantly lower than the half NPP of freely swinging mammalian limbs of similar size (Fig. 2d)^21^. Animals are therefore actively swinging their legs, as has been previously found in African ungulates whose limbs swing faster than if modelled as a pendulum^1^. Faster swing phases than predicted for a compound pendulum are also observed in humans and may be associated with a ballistic model in which muscles are active early in swing to propel the leg forward in an otherwise passive manner^22^. Our findings suggest that such a ballistic model of walking may be broadly applicable. Although swinging the limb at a rate faster than its NPP may be energetically costly, this cost may be mitigated by the geometric advantages of a smoother centre of mass path achieved with relatively shorter steps^23^. Although *t*_swing_ values don’t match the expected NPP values for limbs, the *f*_stride_ data for both mammals and birds are in close agreement with the values of Pennycuick^3^ and are proportional to the natural frequency of a simple pendulum with a length of one third of shoulder height (Fig. 2b). Care must be taken, however, in interpreting these data as in reality, limb morphology is complex and diverse. More data regarding the inertial and mass properties of the limbs of birds and mammals would allow refinement of the model, improving its predictive power.

With the exception of DF, the kinematics of both birds and mammals change with the same exponent of *M*_b_ when walking at self-selected speeds. This suggests commonalities in the underlying mechanics driving preferred gaits regardless of the number of legs. Throughout the range of *M*_b_, however, birds have a higher *t*_stance_ than mammals at their self-selected walking speed. *t*_swing_ is similar between the two classes resulting in higher DF and lower *f*_stride_ in birds. Higher DFs in birds are consistent with their more crouched posture, resulting from their compliant limb morphology. The energetic consequences of these kinematic differences between quadrupeds and bipeds at their preferred speeds are unknown. From DF alone the present data suggest that birds would have a lower CoT at their self-selected speed compared to mammals of the same *M*_b_ as a result of increased foot contact time. Birds, however, may use 1.7 times more energy than quadrupeds for a given rate of force generation, making energetic predictions problematic^24^.

In summary, the hindlimb kinematics of animals walking at self-selected speeds suggest that larger animals (both extant and extinct) prefer to walk relatively more slowly and not at equal values of Fr. Thus, despite being a central tenet of terrestrial locomotor research, Fr and dynamic similarity cannot explain the movement of birds or mammals in the wild. As suggested previously, at their self-selected walking speed, animals walk with *t*_swing_ faster than expected if the limbs were swinging as a simple pendulum. No animals choose to walk at their theoretical minimum DF (0.5) at which the cost of transport reaches its minimum value, indicating energy expenditure may not be the only driver of preferred walking speed. Finally, collecting video data across a broad range of animals is time consuming. Extracting data from web based video footage has limitations (for example, controlling camera orientation relative to the subject and a lack of a scale), yet, nonetheless, this ‘crowdsourcing’ approach to data collection offers a useful tool for the study of animal locomotion outside of the lab, which will no doubt improve in parallel with the camera technology available to the public.

## Methods

### Data collection

Videos of 112 walking bird (n = 32) and mammal species (n = 80) spanning 14 orders (*M*_b_ = 0.61 to 5400 kg) from wild and captive (zoo) individuals were found on YouTube (the entire list of species and video sources can be found in Supplementary Table S1). The kinematic parameters of DF, *f*_stride_, *t*_stance_ and *t*_swing_ were calculated from the relative durations of the stance and swing phases of a single limb using Tracker 4.85^©^ video analysis software (Open Source Physics, Java framework). None of these measures require, either the image scale or the exact angle of the camera lens relative to the animal to be known. The criteria for inclusion in the analyses were that animals were walking whilst not feeding or ground foraging. Strides were analysed for between 1 and 5 individuals per species and for between 2 and 20 strides per individual depending on the availability and length of usable video data. *M*_b_ for all individuals were estimated using published values. In some cases it was possible to determine an individual’s sex. Where sex was indeterminate, the species’ median *M*_b_ was used.

### Statistical analyses

Data were normally distributed (by investigation with quantile-quantile plots and Shapiro-Wilk tests). Analysis of covariance (ANCOVA, two-sided, with a significance level of p < 0.05) was performed on log-transformed data to identify differences in the observed walking kinematics between class groups across all body masses. If the interaction term (class group x *M*_b_) was non-significant indicating similar slopes, they were removed from the ANCOVA model and the test was rerun assuming parallel slopes (testing for differences in the intercepts only. All statistics were conducted in Matlab R2013a (The MathWorks, Inc., Natick, MA, USA). The lines of best fit were calculated from the coefficients table generated by the ANCOVA. The ANCOVA outputs are reported in Tables 2 and 3 of the Supplementary Information.

## Additional Information

**How to cite this article**: Lees, J. *et al.* Locomotor preferences in terrestrial vertebrates: An online crowdsourcing approach to data collection. *Sci. Rep.* **6**, 28825; doi: 10.1038/srep28825 (2016).

## Supplementary Material

Supplementary Information

## Figures and Tables

**Table 1:** Summary of the ANCOVA analysis describing the variation in log transformed kinematic parameters accounted for by group (bird and mammal) and body mass (*M*_b_).

Parameter	Source	Full model			Minimum adequate model				
		*d.f.*	*r*^2^	*F*	*P*	*d.f.*	*r*^2^	*F*	*P*
ln(DF)	group	1	0.2	90.0	0.00	–	–	–	–
	ln(*M*_b_)	1	0.2	59.3	0.00	–	–	–	–
	group-ln(*M*_b_)	1	0.0	5.1	0.02	–	–	–	–
	error	225	0.6						
ln(*f*_stride_)	group	1	0.1	33.9	0.00	1	0.1	34.0	1.9x10^−8^
	ln(*M*_b_)	1	0.5	226.6	0.00	1	0.5	227.5	4.9–10^−36^
	group-ln(*M*_b_)	1	4–10^−5^	0.0	0.89	–	–	–	–
	error	225	0.5			226	0.5		
ln(*t*_stance_)	group	1	0.1	51.8	0.00	1	0.1	52.0	8.3–10^−12^
	ln(*M*_b_)	1	0.5	229.5	0.00	1	0.5	230.4	2.4–10^−36^
	group-ln(*M*_b_)	1	2–10^−4^	0.1	0.74	–	–	–	–
	error	225	0.4			226	0.4		
ln(*t*_swing_)	group	1	2–10^−3^	0.8	0.37	1	2–10^−3^	0.8	0.4
	ln(*M*_b_)	1	0.4	145.8	0.00	1	0.4	143.4	0.0
	group-ln(*M*_b_)	1	0.0	3.9	0.05	–	–	–	–
	error	225	0.6			226	0.6		

The kinematic parameters are duty factor (DF), stride frequency (*f*_stride_, Hz), stance duration (*t*_stance_, s) and swing duration (*t*_swing_, s).

## References

[b1] Dagg A. I. The walking gaits of some species of Pecora. J. Zool. 155, 103–110 (1958).

[b2] Dagg A. I. The walk of the silver gull (*Larus novaehollandiae*) and of other birds. J. Zool. 182, 529–540 (1977).

[b3] Pennycuick C. J. On the running of the Gnu (*Connochaetes taurinus*) and other animals. J. Exp. Biol. 63, 775–799 (1975).

[b4] Alexander R. M. & Jayes A. S. A dynamic similarity hypothesis for the gaits of quadrupedal mammals. J. Zool. 201, 135–152 (1983).

[b5] Jenkins F. A. Limb posture and locomotion in the Virginia opossum (*Didelphis marsupialis*) and in other non-cursorial mammals. J Zool Lond 165, 303–315 (1971).

[b6] Usherwood J. R. Constraints on muscle performance provide a novel explanation for the scaling of posture in terrestrial animals. Biol. Lett. 9, doi: 10.1098/Rsbl.2013.0414 (2013). PMC3730652 23825086

[b7] Gatesy S. M. & Biewener A. A. Bipedal locomotion: effects of speed, size and limb posture in birds and humans. J. Zool. 224, 127–147 (1991).

[b8] Day L. M. & Jayne B. C. Interspecific scaling of the morphology and posture of the limbs during the locomotion of cats (Felidae). J. Exp. Biol. 210, 642–654, doi: 10.1242/Jeb.02703 (2007). 17267650

[b9] Fujita M. Kinematic parameters of the walking of herons, ground-feeders, and waterfowl. Comp. Biochem. Physiol. A. Mol. Integr. Physiol. 139, 117–124, doi: 10.1016/J.Cbpb.2004.07.005 (2004). 15471689

[b10] Taylor C. R., Heglund N. C. & Maloiy G. M. O. Energetics and mechanics of terrestrial locomotion I. Metabolic energy consumption as a function of speed and body size in birds and mammals. J. Exp. Biol. 97, 1–21 (1982). 7086334 10.1242/jeb.97.1.1

[b11] Leurs F. *et al.* Optimal walking speed following changes in limb geometry. J. Exp. Biol. 214, 2276–2282, doi: Doi 10.1242/Jeb.054452 (2011). 21653821

[b12] Pontzer H. Effective limb length and the scaling of locomotor cost in terrestrial animals. J. Exp. Biol. 210, 1752–1761, doi: 10.1242/Jeb.002246 (2007). 17488938

[b13] Halsey L. G. The relationship between energy expenditure and speed during pedestrian locomotion in birds: A morphological basis for the elevated y-intercept ? Comp. Biochem. Physiol., A: Mol. Integr. Physiol. 165, 295–298, doi: Doi 10.1016/J.Cbpa.2013.03.027 (2013). 23545444

[b14] Watson R. R. *et al.* Gait-specific energetics contributes to economical walking and running in emus and ostriches. Proc. R. Soc. B 278, 2040–2046, doi: 10.1098/rspb.2010.2022 (2011). PMC3107644 21123267

[b15] Hoyt D. F. & Taylor R. Gait and the energetics of locomotion in horses. Nature 292, 239–240 (1981).

[b16] MacDougall H. G. & Moore S. T. Marching to the beat of the same drummer: the spontaneous tempo of human locomotion. J. Appl. Physiol. 99, 1164–1173, doi: 10.1152/japplphysiol.00138.2005 (2005). 15890757

[b17] Raichlen D. A., Pontzer H. & Shapiro L. J. A new look at the Dynamic Similarity Hypothesis: the importance of swing phase. Biol. Open. 2, 1032–1036, doi: Doi 10.1242/Bio.20135165 (2013). 24167713 PMC3798186

[b18] Tickle P. G., Richardson M. F. & Codd J. R. Load carrying during locomotion in the barnacle goose (*Branta leucopsis*): The effect of load placement and size. Comp. Biochem. Physiol. A. Mol. Integr. Physiol. 156, 309–317, doi: 10.1016/j.cbpa.2010.01.022 (2010). 20153444

[b19] Marsh R. L., Ellerby D. J., Carr J. A., Henry H. T. & Buchanan C. I. Partitioning the energetics of walking and running: swinging the limbs is expensive. Science 303, 80–83, doi: 10.1126/science.1090704 (2004). 14704426

[b20] Usherwood J. R., Szymanek K. L. & Daley M. A. Compass gait mechanics account for top walking speeds in ducks and humans. J. Exp. Biol. 211, 3744–3749, doi: 10.1242/Jeb.023416 (2008). 19011215 PMC2978950

[b21] Kilbourne B. M. & Hoffman L. C. Scale effects between body size and limb design in quadrupedal mammals. Plos One 8, doi: 10.1371/journal.pone.0078392 (2013). PMC3832634 24260117

[b22] Mochon S. & Mcmahon T. A. Ballistic walking. J. Biomech. 13, 49–57, doi: 10.1016/0021-9290(80)90007-X (1980). 7354094

[b23] Kuo A. D. Energetics of actively powered locomotion using the simplest walking model. J. Biomech. Eng. Trans. ASME 124, 113–120, doi: 10.1115/1.1427703 (2002). 11871597

[b24] Roberts T. J., Kram R., Weyand P. G. & Taylor C. R. Energetics of bipedal running I. Metabolic cost of generating force. J. Exp. Biol. 201, 2745–2751 (1998). 9732329 10.1242/jeb.201.19.2745

