# Supplementary material for: Locomotor preferences in terrestrial vertebrates: An online crowdsourcing approach to data collection
Source: Sci Rep. 2016 Jul 6;6:28825. doi: 10.1038/srep28825 (PMC4933880; doi:10.1038/srep28825)
Supplement: Supplementary Information [file srep28825-s1.pdf]

Supplementary Information:

Locomotor preferences in terrestrial vertebrates: An online crowdsourcing approach to data collection

John Lees[1]*, James Gardiner[2], Jim Usherwood[3], Robert Nudds[1]

[1]Faculty of Life Sciences, University of Manchester, Manchester, M13 9PT, UK
[2] School of Computing, Science and Engineering, University of Salford, Salford, M5 4WT, UK
[3] Structure and Motion Laboratory, The Royal Veterinary College, University of London, North Mymms, Hatfield, Herts, AL9 7TA, UK
*Author for correspondence: John Lees, John.Lees@manchester.ac.uk

Supplementary Table S1: Summary of the species used in the study, their body mass estimates, kinematic parameters and the videos from which they were calculated.

Species name	Class	Order	Genus	Species	URL	Video title	Body mass (kg)	Body mass source	Stride frequency (Hz)	Duty factor	Stance duration (s)	Swing duration (s)
Bengal tiger	Mammalia	Carnivora	*Panthera*	*tigris tigris*	https://www.youtube.com/watch?v=S0wnvyxIOx0	royal Bengal tiger walking	169	[1]	0.62	0.69	1.12	0.50
Bengal tiger	Mammalia	Carnivora	*Panthera*	*tigris tigris*	https://www.youtube.com/watch?v=b7zYcZ-s-Os	tiger vs lion walking	169	[1]	0.69	0.65	0.94	0.50
Canada lynx	Mammalia	Carnivora	*Lynx*	*canadensis*	https://www.youtube.com/watch?v=QTP2r-0dTq4	lynx walk-by	11	[1]	1.05	0.66	0.63	0.33
Ocelot	Mammalia	Carnivora	*Leopardus*	*pardalis*	https://www.youtube.com/watch?v=byG1JQYHCJ0	ocelot at woodland park zoo Seattle	9.4	[1]	1.05	0.61	0.58	0.37
Ocelot	Mammalia	Carnivora	*Leopardus*	*pardalis*	https://www.youtube.com/watch?v=1d8Ec4Y-91g	ocelot walking around	9.4	[1]	1.22	0.61	0.50	0.32
Ocelot	Mammalia	Carnivora	*Leopardus*	*pardalis*	https://www.youtube.com/watch?v=jgv1CHINSAc	fauna forever camera trap research by volunteers reveals interesting ocelot footage	9.4	[1]	1.32	0.58	0.44	0.32
Ocelot	Mammalia	Carnivora	*Leopardus*	*pardalis*	https://www.youtube.com/watch?v=jgv1CHINSAc	fauna forever camera trap research by volunteers reveals interesting ocelot footage	9.4	[1]	1.01	0.61	0.60	0.39
Leopard	Mammalia	Carnivora	*Panthera*	*pardus*	https://www.	Leopard walking on	39	[1]	1.01	0.67	0.66	0.33

					youtube.com /watch?v=zTT2i6KjiOM	road in Kruger National Park.						
Leopard	Mammalia	Carnivora	*Panthera*	*pardus*	https://www.youtube.com/watch?v=GyrxUdqa_pI	Samburu Kenya - Leopard walking near our car	39	[1]	1.07	0.64	0.60	0.34
Lion	Mammalia	Carnivora	*Panthera*	*leo*	https://www.youtube.com/watch?v=o7oPKchbYbk	male lion walking	167	[1]	0.70	0.68	0.98	0.45
Lion	Mammalia	Carnivora	*Panthera*	*leo*	https://www.youtube.com/watch?v=1eB7vv1J9F8	Free Stock Footage Male Lion Walking on a Dusty Plain - Africa Travel Channel in HD	167	[1]	0.82	0.65	0.80	0.43
Lion	Mammalia	Carnivora	*Panthera*	*leo*	https://www.youtube.com/watch?v=moEAUPLB2G0	Lion Walking along the ridge : Awesome Capture!	167	[1]	0.75	0.68	0.91	0.42
Lion	Mammalia	Carnivora	*Panthera*	*leo*	https://www.youtube.com/watch?v=Kc3kGyqwQDc	African lion walking between cars safari	167	[1]	0.71	0.71	1.00	0.42
Lion	Mammalia	Carnivora	*Panthera*	*leo*	https://www.youtube.com/watch?v=-KwBP_h8ToQ	Male lion walking out of woods to join pride	167	[1]	0.91	0.71	0.77	0.32
Cheetah	Mammalia	Carnivora	*Acinonyx*	*jubatus*	https://www.youtube.com/watch?v=nmpY1L4-e_g	mashatu - cheetah walking	48	[1]	0.81	0.64	0.78	0.45
Cheetah	Mammalia	Carnivora	*Acinonyx*	*jubatus*	https://www.youtube.com/watch?v=XYYU1XNupj0	cheetah walking	48	[1]	0.90	0.68	0.75	0.36

Serval	Mammalia	Carnivora	*Leptailurus*	*serval*	https://www.youtube.com/watch?v=k03mrE9cm_k	serval.mp4	8.5	[1]	0.88	0.69	0.79	0.35
Caracle	Mammalia	Carnivora	*Caracal*	*caracal*	https://www.youtube.com/watch?v=Q7WXajobrTs	caracal!!!	11.75	[2]	1.10	0.66	0.60	0.31
Caracle	Mammalia	Carnivora	*Caracal*	*caracal*	https://www.youtube.com/watch?v=nsPxt9eudB8	caracle	11.75	[2]	1.11	0.60	0.54	0.36
Cougar	Mammalia	Carnivora	*Puma*	*concolor*	https://www.youtube.com/watch?v=akAU5fhPl5M	a pair of cougars walking	60	[1]	0.82	0.68	0.83	0.38
Cougar	Mammalia	Carnivora	*Puma*	*concolor*	https://www.youtube.com/watch?v=TKoQmx01LFY	Cougar - capitol forest	60	[1]	0.77	0.65	0.85	0.45
Domestic cat	Mammalia	Carnivora	*Felis*	*silvestris catus*	https://www.youtube.com/watch?v=Hzt18V3Uaxc	cat walking	3.7	[1]	1.03	0.67	0.65	0.32
Leopard	Mammalia	Carnivora	*Panthera*	*pardus*	https://www.youtube.com/watch?v=DkMqWTebg4M	Leopard walk	39	[1]	0.90	0.68	0.75	0.36
Cheetah	Mammalia	Carnivora	*Acinonyx*	*jubatus*	https://www.youtube.com/watch?v=NW-AhFU6sT4	Cheetah walking	48	[1]	0.97	0.63	0.65	0.38
African civet	Mammalia	Carnivora	*Civettictis*	*civetta*	https://www.youtube.com/watch?v=Hy	African civet cat walking at night. Niassa Reserve,	13.5	[2]	1.59	0.65	0.41	0.22

					590z-1TfQ	Mozambique.. Stock Footage						
Gorilla	Mammalia	Primates	*Gorilla*	*gorilla*	https://www.youtube.com/watch?v=2Npc5QIS6Iw	Gorilla walking	169	[3]	0.59	0.63	1.08	0.62
Gorilla	Mammalia	Primates	*Gorilla*	*gorilla*	https://www.youtube.com/watch?v=2Npc5QIS6Iw	Gorilla walking	169	[3]	0.60	0.62	1.04	0.63
Gorilla	Mammalia	Primates	*Gorilla*	*gorilla*	https://www.youtube.com/watch?v=8sGkN6YCylc	Gorilla walking	169	[3]	0.70	0.67	0.97	0.47
Gorilla	Mammalia	Primates	*Gorilla*	*gorilla*	https://www.youtube.com/watch?v=IHi3xYS3cOs	Silverback gorilla walking next to camera	169	[3]	0.64	0.65	1.03	0.55
Chimpanzee	Mammalia	Primates	*Pan*	*troglodytes*	https://www.youtube.com/watch?v=zTZs_ymXZm8	Chimpanzee knuckle walking	50	[3]	0.78	0.66	0.84	0.43
Rhesus macaque	Mammalia	Primates	*Macaca*	*mulatta*	https://www.youtube.com/watch?v=AocYAYgyWp0	Macacques walking about in a city	9.9	[3]	1.01	0.59	0.58	0.41
Japanese macaque	Mammalia	Primates	*Macaca*	*fuscata*	https://www.youtube.com/watch?v=yze3og2dKzw	Japanese macacque locomotion 1	9.5	[3]	0.87	0.69	0.79	0.36
Rhesus macaque	Mammalia	Primates	*Macaca*	*mulatta*	https://www.youtube.com/watch?v=fw7JBfxGoCg	wild macaque walking along road	9.9	[3]	0.94	0.64	0.68	0.38
Olive baboon	Mammalia	Primates	*Papio*	*anubis*	https://www.youtube.com/watch?v=M	baboon walking	25.1	[3]	1.01	0.58	0.58	0.41

					Ye61EN1vLU							
Vervet monkey	Mammalia	Primates	*Chlorocebus*	*pygerythrus*	https://www.youtube.com/watch?v=4ZXpJ0i9W_I	olive baboon at salt lick game lodge	7	[4]	1.21	0.62	0.52	0.31
Yellow baboon	Mammalia	Primates	*Papio*	*cynocephalus*	https://www.youtube.com/watch?v=WQf748auQLk	A Yellow Baboon on the Road in Tanzania, Africa	17.05	[3]	0.84	0.60	0.71	0.48
Polar bear	Mammalia	Carnivora	*Ursus*	*maritimus*	https://www.youtube.com/watch?v=TQDDl0OIfsU	Polar bear walk, jump, swim - Ours polaire se prom ne ,saute et nage	217	[5]	0.82	0.64	0.78	0.44
Polar bear	Mammalia	Carnivora	*Ursus*	*maritimus*	https://www.youtube.com/watch?v=URWsoBdgsgQ	polar bear walking	217	[5]	0.68	0.68	1.00	0.48
Grizzly bear	Mammalia	Carnivora	*Ursus*	*arctos*	https://www.youtube.com/watch?v=5LabWRzjlf4&list=UUcfQVMHFyx1Ziy1ks6pCRZQ	Grizzly Bear Walking (Slow Motion Animation Reference)	163.9	[6]	0.68	0.70	1.03	0.45
Black bear	Mammalia	Carnivora	*Ursus*	*americanus*	https://www.youtube.com/watch?v=tE2GxrSgd7I	Black Bear going after a grouse next to the road	100	[7]	0.76	0.69	0.92	0.41
Spectacled bear	Mammalia	Carnivora	*Tremarctos*	*ornatus*	https://www.youtube.com/watch?v=uT54Ht-3HuE	Homework Hotline: Zoophilly: Spectacle Bear	100	[8]	0.81	0.70	0.87	0.37
Spectacled bear	Mammalia	Carnivora	*Tremarctos*	*ornatus*	https://www.youtube.com/watch?v=5b	Philadelphia Zoo Spectacled Bear Heading for His	100	[8]	0.74	0.75	1.02	0.34

					usidmX434	Favorite Place						
Sun bear	Mammalia	Carnivora	*Helarctos*	*malayanus*	https://www.youtube.com/watch?v=S9HG1NpoLnA	Sun Bear Merapoh	34.7	[9]	0.66	0.66	1.00	0.52
Giant panda	Mammalia	Carnivora	*Ailuropoda*	*melanoleuca*	https://www.youtube.com/watch?v=fy-_J5HRlr0	Giant Panda Walking Around	87.50	[10]	0.74	0.68	0.92	0.43
Algarvia goat	Mammalia	Artiodactyla	*Capra*	*hircus*	https://www.youtube.com/watch?v=TjLGDYBnHCI	Algarvia goats (Capra hircus), Porches, Algarve, Portugal, Europe	60	[11]	1.28	0.60	0.47	0.31
Giraffe	Mammalia	Artiodactyla	*Giraffa*	*camelopardalis*	https://www.youtube.com/watch?v=TiGl5VLmh00	a giraffe walking around	1340	[2]	0.41	0.65	1.57	0.85
Giraffe	Mammalia	Artiodactyla	*Giraffa*	*camelopardalis*	https://www.youtube.com/watch?v=R0uNbM0dADc	giraffe running in herd of cattle, Kenya	1340	[2]	0.51	0.60	1.17	0.78
Giraffe	Mammalia	Artiodactyla	*Giraffa*	*camelopardalis*	https://www.youtube.com/watch?v=be7RtceomL0	giraffe walk	1340	[2]	0.41	0.70	1.68	0.74
Giraffe	Mammalia	Artiodactyla	*Giraffa*	*camelopardalis*	https://www.youtube.com/watch?v=vB_r-Oi6_yk	giraffe walking around in 1080p HD	1340	[2]	0.49	0.67	1.38	0.68
Dromedary camel	Mammalia	Artiodactyla	*Camelus*	*dromedarius*	https://www.youtube.com/watch?v=eCmcX-7v6LY	dromedary camel	400.8	[12]	0.56	0.68	1.23	0.57
Dromedary camel	Mammalia	Artiodactyla	*Camelus*	*dromedarius*	https://www.youtube.com/watch?v=Svt5BpwxAfk	the dromedary camel camelus dromedarius one hump	400.8	[12]	0.55	0.69	1.24	0.56

White tailed deer	Mammalia	Artiodactyla	*Odocoileus*	*viginianus*	https://www.youtube.com/watch?v=bvYReWsOe6Q	two deer walking around town	100	[13]	0.79	0.71	0.90	0.37
Moose	Mammalia	Artiodactyla	*Alces*	*alces*	https://www.youtube.com/watch?v=Po6vTQT9l3s	moose walking through prairie farm yard	450	[13]	0.67	0.69	1.02	0.46
Moose	Mammalia	Artiodactyla	*Alces*	*alces*	https://www.youtube.com/watch?v=mHHXSGAv3P4	Smithers B.C. moose walking down the street3384.AVI	450	[13]	0.62	0.70	1.13	0.48
Moose	Mammalia	Artiodactyla	*Alces*	*alces*	https://www.youtube.com/watch?v=ClsPNKljLuE	Bull moose walking across the road	450	[13]	0.69	0.70	1.01	0.43
Hippo	Mammalia	Artiodactyla	*Hippopotamus*	*amphibius*	https://www.youtube.com/watch?v=ej6DrFpnJrk	long walk for a hippo	3800	[13]	0.50	0.74	1.48	0.52
Hippo	Mammalia	Artiodactyla	*Hippopotamus*	*amphibius*	https://www.youtube.com/watch?v=SBCy2kSb-WQ	hippo walking away - Zambia safari 2007	3800	[13]	0.65	0.73	1.12	0.41
Blue wildebeest	Mammalia	Artiodactyla	*Connochaetes*	*taurinus*	https://www.youtube.com/watch?v=JMOztPyARno	wildebeest in hd	250	[13]	0.71	0.69	0.97	0.45
Pronghorn antelope	Mammalia	Artiodactyla	*Antilocapra*	*americana*	https://www.youtube.com/watch?v=5aNYPf3wB5c	antelope on the Gallatin river ranch	50	[13]	0.98	0.65	0.67	0.35
Gerenuk	Mammalia	Artiodactyla	*Litocranius*	*walleri*	https://www.youtube.com/watch?v=F2	Gerenuks - Waller's gazelle - Giraffe-necked Antelope.	39	[2]	0.79	0.66	0.83	0.43

					udbhZg1tA	Oregon Zoo						
Impala	Mammalia	Artiodactyla	*Aepyceros*	*melampus*	https://www.youtube.com/watch?v=vbun7hLsFTM	2013-01-08 Banded Mongoose walking with Impala, Chobe NP, Botswana	50	[13]	0.86	0.62	0.72	0.44
Greater kudu	Mammalia	Artiodactyla	*Tragelaphus*	*strepsiceros*	https://www.youtube.com/watch?v=XIQUqfiox7s	kudu walk in front of my car	167.5	[2]	0.62	0.68	1.09	0.51
Greater kudu	Mammalia	Artiodactyla	*Tragelaphus*	*strepsiceros*	https://www.youtube.com/watch?v=6PpAIqPAWFE	majestic kudu in Krugerpark	252.5	[2]	0.63	0.63	1.00	0.58
Llama	Mammalia	Artiodactyla	*Lama*	*glama*	https://www.youtube.com/watch?v=iEqqlhAtSQA&list=UUcfQVMHFyx1Ziy1ks6pCRZQ	Llama Walking (Slow Motion Animation Reference)	142.5	[11]	0.68	0.67	0.99	0.49
Pronghorn antelope	Mammalia	Artiodactyla	*Antilocapra*	*americana*	https://www.youtube.com/watch?v=LYZP7pru7Lg	Hunt for More #5 Pronghorn Buck walks past truck	50	[13]	1.06	0.60	0.57	0.38
Eland	Mammalia	Artiodactyla	*Taurotragus*	*oryx*	https://www.youtube.com/watch?v=NoBPI25fgrs	Eland at Klopperfontein waterhole Kruger Park	900	[13]	0.59	0.65	1.10	0.59
Eland	Mammalia	Artiodactyla	*Taurotragus*	*oryx*	https://www.youtube.com/watch?v=NoBPI25fgrs	Eland at Klopperfontein waterhole Kruger Park	900	[13]	0.58	0.67	1.15	0.57
Eland	Mammalia	Artiodactyla	*Taurotragus*	*oryx*	https://www.youtube.com/watch?v=NoBPI25fgrs	Eland at Klopperfontein waterhole Kruger Park	900	[13]	0.60	0.67	1.10	0.55
Eland	Mammalia	Artiodactyl	*Taurotragu*	*oryx*	https://www.	A walking eland bull	900	[13]	0.53	0.71	1.36	0.54

		a	*s*		youtube.com/watch?v=wXqMRgoz0sQ	knee-clicking. [Stock footage]						
American bison	Mammalia	Artiodactyla	*Bison*	*bison*	https://www.youtube.com/watch?v=eEYg4GMiJ4w	Yellowstone Bison Walking Down The Middle Of The Road	900	[13]	0.69	0.68	1.00	0.46
American bison	Mammalia	Artiodactyla	*Bison*	*bison*	https://www.youtube.com/watch?v=7nbDEloMQiI	Bison walking through Yellowstone traffic	900	[13]	0.75	0.67	0.90	0.44
American bison	Mammalia	Artiodactyla	*Bison*	*bison*	https://www.youtube.com/watch?v=f35Ui78BzII	Bison walking down the road in Yellowstone NP	900	[13]	0.66	0.70	1.06	0.46
African buffalo	Mammalia	Artiodactyla	*Syncerus*	*caffer*	https://www.youtube.com/watch?v=KZ52NAWwhtA	Cape Buffalo Herd (with baby)	750	[13]	0.64	0.68	1.06	0.50
African buffalo	Mammalia	Artiodactyla	*Syncerus*	*caffer*	https://www.youtube.com/watch?v=KZ52NAWwhtA	Cape Buffalo Herd (with baby)	750	[13]	0.79	0.66	0.84	0.43
African buffalo	Mammalia	Artiodactyla	*Syncerus*	*caffer*	https://www.youtube.com/watch?v=KZ52NAWwhtA	Cape Buffalo Herd (with baby)	750	[13]	0.69	0.66	0.96	0.49
African buffalo	Mammalia	Artiodactyla	*Syncerus*	*caffer*	https://www.youtube.com/watch?v=kMwBAObIePk	Hundreds of Cape Buffalo Ulusaba	750	[13]	0.77	0.64	0.83	0.47
Red deer	Mammalia	Artiodactyla	*Cervus*	*elaphus*	https://www.youtube.com/watch?v=MD97N71ZGjc	Two rival Red Deer Stags (Royals)	300	[13]	0.49	0.71	1.44	0.59
Red deer	Mammalia	Artiodactyl	*Cervus*	*elaphus*	https://www.	Two rival Red Deer	300	[13]	0.47	0.74	1.56	0.56

		a			youtube.com /watch?v=MD97N71ZGjc	Stags (Royals)						
Red deer	Mammalia	Artiodactyla	*Cervus*	*elaphus*	https://www.youtube.com/watch?v=jhDMIenueJ0	Edelherten imponeren	300	[13]	0.72	0.65	0.90	0.49
Roan antelope	Mammalia	Artiodactyla	*Hippotragus*	*equinus*	https://www.youtube.com/watch?v=rxItEy5UKZ8	Selinda roan antelope	250	[13]	0.67	0.61	0.91	0.57
Greater kudu	Mammalia	Artiodactyla	*Tragelaphus*	*strepsiceros*	https://www.youtube.com/watch?v=k68LVleK-6A	Black faced impala, kudu & hartebeest @ an Etosha NP waterhole	252.5	[2]	0.56	0.69	1.25	0.55
Greater kudu	Mammalia	Artiodactyla	*Tragelaphus*	*strepsiceros*	https://www.youtube.com/watch?v=k68LVleK-6A	Black faced impala, kudu & hartebeest @ an Etosha NP waterhole	252.5	[2]	0.69	0.63	0.90	0.54
Red hartebeest	Mammalia	Artiodactyla	*Alcelaphus*	*caama*	https://www.youtube.com/watch?v=FMlhPo5u2Uo	Zebra-Red Hartebeest-One Gemsbok	170	[13]	0.71	0.68	0.96	0.46
Red hartebeest	Mammalia	Artiodactyla	*Alcelaphus*	*caama*	https://www.youtube.com/watch?v=FMlhPo5u2Uo	Zebra-Red Hartebeest-One Gemsbok	170	[13]	0.77	0.64	0.82	0.47
Bighorn sheep	Mammalia	Artiodactyla	*Ovis*	*canadensis*	https://www.youtube.com/watch?v=zD-M9PAZ4N4	Bighorn Sheep walking by my Jeep	150	[13]	0.96	0.62	0.65	0.39
Bighorn sheep	Mammalia	Artiodactyla	*Ovis*	*canadensis*	https://www.youtube.com/watch?v=VLcB8pxaaBQ	Bighorn Sheep Walking Along the Road in Jasper National Park	150	[13]	1.12	0.63	0.57	0.33
Bighorn sheep	Mammalia	Artiodactyla	*Ovis*	*canadensis*	https://www.youtube.com	watching the bighorn sheep	150	[13]	0.77	0.69	0.90	0.40

					/watch?v=MQNJnLnA-iw							
Bighorn sheep	Mammalia	Artiodactyla	*Ovis*	*canadensis*	https://www.youtube.com/watch?v=MQNJnLnA-iw	watching the bighorn sheep	150	[13]	0.85	0.63	0.75	0.44
Bighorn sheep	Mammalia	Artiodactyla	*Ovis*	*canadensis*	https://www.youtube.com/watch?v=2Jua0hrIHBA	Canadian Rockies, Banff - A Big Horn Sheep walking in the parking lot	150	[13]	1.22	0.63	0.51	0.30
Topi	Mammalia	Artiodactyla	*Damaliscus*	*korrigum*	https://www.youtube.com/watch?v=l-seaz09i-E	Topi walking away in Mburo National Park	130	[13]	0.83	0.63	0.76	0.44
Reindeer	Mammalia	Artiodactyla	*Rangifer*	*tarandus*	https://www.youtube.com/watch?v=3vLbF_VgQMc	Britain's only herd of wild Reindeer - at Ben Macdui, Ballater, Aberdeenshire	120	[13]	0.84	0.70	0.83	0.36
Reindeer	Mammalia	Artiodactyla	*Rangifer*	*tarandus*	https://www.youtube.com/watch?v=3vLbF_VgQMc	Britain's only herd of wild Reindeer- at Ben Macdui, Ballater, Aberdeenshire	120	[13]	0.94	0.68	0.72	0.34
White tailed deer	Mammalia	Artiodactyla	*Odocoileus*	*viginianus*	https://www.youtube.com/watch?v=_bVbG1lj9Yk	Backyard whitetail deer in Bozeman	100	[13]	0.95	0.64	0.68	0.37
White tailed deer	Mammalia	Artiodactyla	*Odocoileus*	*viginianus*	https://www.youtube.com/watch?v=6WDlkk1cDPU	Close Encounter with a wild Whitetail Deer	100	[13]	0.78	0.67	0.86	0.42
Tibetan argali	Mammalia	Artiodactyla	*Ovis*	*ammon*	https://www.youtube.com/watch?v=j-VQ8WEPEwg	Tibetan argali (Ovis ammon hodgsonii) sheep grazing in Ladakh	65	[13]	0.96	0.67	0.70	0.34
Tibetan	Mammalia	Artiodactyl	*Ovis*	*ammon*	https://www.	Tibetan argali (Ovis	65	[13]	0.90	0.67	0.74	0.37

argali		a			youtube.com /watch?v=j-VQ8WEPEwg	ammon hodgsonii) sheep grazing in Ladakh						
Grant's gazelle	Mammalia	Artiodactyla	*Nanger*	*granti*	https://www.youtube.com/watch?v=iAjxWzxc150	Grant's gazelle	62	[13]	0.99	0.63	0.63	0.37
Roe deer	Mammalia	Artiodactyla	*Capreolus*	*capreolus*	https://www.youtube.com/watch?v=2_-MIABD5s4	Roe deer CANON EOS 550D CROP MODE.	50	[13]	0.94	0.65	0.70	0.37
Blackbuck	Mammalia	Artiodactyla	*Antilope*	*cervicapra*	https://www.youtube.com/watch?v=DPJ-VhbeUd0	Blackbuck herd within Sikandra Fort premises	37	[13]	0.85	0.74	0.87	0.31
Blackbuck	Mammalia	Artiodactyla	*Antilope*	*cervicapra*	https://www.youtube.com/watch?v=MdBJEhzpTI8	Black buck in Velavadar Black Buck National Park	37	[13]	1.08	0.64	0.60	0.33
Blackbuck	Mammalia	Artiodactyla	*Antilope*	*cervicapra*	https://www.youtube.com/watch?v=y30ob337jRc	Blackbuck at Talchappar Sanctuary in Rajasthan	37	[13]	0.96	0.63	0.66	0.38
Thompson's gazelle	Mammalia	Artiodactyla	*Eudorcas*	*thomsonii*	https://www.youtube.com/watch?v=G7D2kVQFqn8	Thompson's Gazelles Grazing	20	[13]	1.04	0.66	0.64	0.32
Common warthog	Mammalia	Artiodactyla	*Phacochoerus*	*africanus*	https://www.youtube.com/watch?v=X1VGd_YAi4Q	Warthogs walking - Kruger National Park	82.5	[2]	1.43	0.64	0.45	0.25
Common warthog	Mammalia	Artiodactyla	*Phacochoerus*	*africanus*	https://www.youtube.com/watch?v=f6A1JoJ71UM	warthog walking around	82.5	[2]	0.96	0.71	0.74	0.30
Wild	Mammalia	Artiodactyl	*Sus*	*scrofa*	https://www.	wild boar	63.1	[14]	0.86	0.70	0.81	0.35

boar		a			youtube.com/watch?v=Q-4gsMTZwOw							
Hippo	Mammalia	Artiodactyla	*Hippopotamus*	*amphibius*	https://www.youtube.com/watch?v=CIc2k6zY3mI	do the hippo walk	3800	[13]	0.60	0.73	1.22	0.45
Porcupine	Mammalia	Rodentia	*Erethizon*	*dorsatum*	https://www.youtube.com/watch?v=YZRIoZv9BpU	Porcupine scratching and walking at the Alaska Zoo, Anchorage	10.75	[15]	1.04	0.66	0.63	0.32
Capybara	Mammalia	Rodentia	*Hydrochoerus*	*hydrochaeris*	https://www.youtube.com/watch?v=BVro95PvgS8	capybara walking	48.9	[16]	0.56	0.77	1.37	0.41
Capybara	Mammalia	Rodentia	*Hydrochoerus*	*hydrochaeris*	https://www.youtube.com/watch?v=YGg37vzXDhg	Reference: Capybara Walking	48.9	[16]	0.58	0.72	1.25	0.48
Capybara	Mammalia	Rodentia	*Hydrochoerus*	*hydrochaeris*	https://www.youtube.com/watch?v=69hhYfxaR0A	Reference: Capybara (1)	48.9	[16]	0.64	0.75	1.17	0.40
Asian elephant	Mammalia	Proboscidia	*Elephas*	*maximus*	https://www.youtube.com/watch?v=g0xU-gnrh6U	Indian elephant walking and raising its trunk	5400	[11]	0.33	0.69	2.10	0.94
African elephant	Mammalia	Proboscidia	*Loxodontia*	*africana*	https://www.youtube.com/watch?v=rbYTW8i5bXA	African Elephant walking at the Toronto Zoo	5150	[2]	0.25	0.79	3.16	0.86
Asian elephant	Mammalia	Proboscidia	*Elephas*	*maximus*	https://www.youtube.com/watch?v=oLsRNnY5050	Herd of elephants walking in the grassland at Kaziranga	2720	[11]	0.32	0.70	2.17	0.93

African elephant	Mammalia	Proboscidia	*Loxodontia*	*africana*	https://www.youtube.com/watch?v=93BdXvPfPRw	Big Big Big Elephant Walks By Our Car on Safari	5150	[2]	0.39	0.71	1.81	0.75
Asian elephant	Mammalia	Proboscidia	*Elephas*	*maximus*	https://www.youtube.com/watch?v=iOE7dTuLCiU	elephant walking to the shops	5400	[11]	0.41	0.71	1.76	0.71
Asian elephant	Mammalia	Proboscidia	*Elephas*	*maximus*	https://www.youtube.com/watch?v=gTtrW5DLwzA	Elephant & Indian Bison in one Frame.	2720	[11]	0.39	0.67	1.73	0.84
Asian elephant	Mammalia	Proboscidia	*Elephas*	*maximus*	https://www.youtube.com/watch?v=1KG3B9CYHSQ	Asian elephants oregon zoo	2720	[11]	0.36	0.71	1.98	0.81
Spotted hyena	Mammalia	Carnivora	*Crocuta*	*crocuta*	https://www.youtube.com/watch?v=lMX9CS08HyE	Spotted Hyena: Kruger Park, South Africa	65	[13]	1.14	0.64	0.56	0.32
Spotted hyena	Mammalia	Carnivora	*Crocuta*	*crocuta*	https://www.youtube.com/watch?v=Mvsv4OdcT3g	Camp Olkonoi: Hyena Walking	65	[13]	1.15	0.60	0.52	0.35
Spotted hyena	Mammalia	Carnivora	*Crocuta*	*crocuta*	https://www.youtube.com/watch?v=55LNHNHrnYE	Spotted Hyena on Safari in Kruger Park	65	[13]	1.03	0.61	0.59	0.38
Striped hyena	Mammalia	Carnivora	*Hyaena*	*hyaena*	https://www.youtube.com/watch?v=P9YUPy5sSJM	Striped Hyena	40	[2]	1.35	0.62	0.46	0.28
Striped hyena	Mammalia	Carnivora	*Hyaena*	*hyaena*	https://www.youtube.com/watch?v=I1wvg1Q7sVU	Striped Hyenas	40	[2]	0.97	0.67	0.68	0.34

Brown hyena	Mammalia	Carnivora	*Hyaena*	*brunnea*	https://www.youtube.com/watch?v=5yT6f5DDZAs	Brown Hyena.Kgalagadi	47.5	[2]	1.07	0.66	0.61	0.32
Meerkat	Mammalia	Carnivora	*Suricata*	*suricatta*	https://www.youtube.com/watch?v=rpY5eto4aFE	meerkat walking	0.80	[2]	1.49	0.63	0.42	0.25
Baird's tapir	Mammalia	Perissodactyla	*Tapirus*	*bairdii*	https://www.youtube.com/watch?v=g3QdARq5Dm0	Tapir walking near me in 1080P HD by John D. Villarreal	225	[17]	0.64	0.68	1.07	0.50
Baird's tapir	Mammalia	Perissodactyla	*Tapirus*	*bairdii*	https://www.youtube.com/watch?v=EpFkGookteY	A walking tapir	225	[17]	0.57	0.73	1.27	0.48
Indian rhinoceros	Mammalia	Perissodactyla	*Rhinoceros*	*unicornis*	https://www.youtube.com/watch?v=BquiWvSQH0E	Rhinoceros walk cycle	1705	[18]	0.53	0.69	1.31	0.59
Plains Zebra	Mammalia	Perissodactyla	*Equus*	*quagga*	https://www.youtube.com/watch?v=_z5VeEFyBXk	zebra walking	300	[19]	0.86	0.66	0.77	0.40
Plains Zebra	Mammalia	Perissodactyla	*Equus*	*quagga*	https://www.youtube.com/watch?v=k2QWqkwGCE4	Etosha walking (driving) with the zebra	300	[19]	0.79	0.65	0.82	0.45
Plains Zebra	Mammalia	Perissodactyla	*Equus*	*quagga*	https://www.youtube.com/watch?v=pUww5YHyK90	Zebra walking at Calauit Wildlife Sanctuary	300	[19]	0.73	0.66	0.91	0.47
Plains Zebra	Mammalia	Perissodactyla	*Equus*	*quagga*	https://www.youtube.com/watch?v=pU	Zebra walking at Calauit Wildlife Sanctuary	300	[19]	0.78	0.67	0.86	0.42

					ww5YHyK90							
South american tapir	Mammalia	Perissodactyla	*Tapirus*	*terrestris*	https://www.youtube.com/watch?v=2RiSYbdbxVw	Tapirus terrestris, South American Tapir, Fauna, Zoo S‹o Paulo,	200	[20]	0.71	0.68	0.95	0.45
South american tapir	Mammalia	Perissodactyla	*Tapirus*	*terrestris*	https://www.youtube.com/watch?v=6R9DweQLpo4	South American(Brazillian)Tapir to move	200	[20]	0.99	0.65	0.66	0.35
South american tapir	Mammalia	Perissodactyla	*Tapirus*	*terrestris*	https://www.youtube.com/watch?v=hcsAsg8nw-U	Brazilian endangered tapir drinking and walking away in Pantanal	200	[20]	0.85	0.67	0.78	0.39
South american tapir	Mammalia	Perissodactyla	*Tapirus*	*terrestris*	https://www.youtube.com/watch?v=ez70ZunXciQ	Two Brazilian Tapirs In A Field	200	[20]	0.71	0.65	0.92	0.49
Malayan tapir	Mammalia	Perissodactyla	*Tapirus*	*indicus*	https://www.youtube.com/watch?v=ZMCqcIyBuYQ	The Malayan Tapir at the Nurnberg Tiergarten	395	[21]	0.65	0.65	1.01	0.53
Malayan tapir	Mammalia	Perissodactyla	*Tapirus*	*indicus*	https://www.youtube.com/watch?v=7yPellyvlBU	Malayan Tapir at Bronx Zoo	395	[21]	0.74	0.67	0.90	0.45
Malayan tapir	Mammalia	Perissodactyla	*Tapirus*	*indicus*	https://www.youtube.com/watch?v=M1o25beVfYA	Malayan tapir Cry	395	[21]	0.60	0.64	1.06	0.60
South american tapir	Mammalia	Perissodactyla	*Tapirus*	*terrestris*	https://www.youtube.com/watch?v=k83w5etmWnE	Brazilian Tapir Walking	200	[20]	0.82	0.67	0.81	0.40
Black rhinoceros	Mammalia	Perissodactyla	*Diceros*	*bicornis*	https://www.youtube.com/watch?v=xN	black rhino Walks across the road	1050	[2]	0.58	0.71	1.22	0.51

					PTO82r3Bc							
Black rhinoceros	Mammalia	Perissodactyla	*Diceros*	*bicornis*	https://www.youtube.com/watch?v=JFFyGpHegAQ	Black Rhino and her calf walking up from the River - Zimbabwe	1050	[2]	0.54	0.70	1.28	0.56
Black rhinoceros	Mammalia	Perissodactyla	*Diceros*	*bicornis*	https://www.youtube.com/watch?v=2u8c0TLPOHo	Rhino walking in the Kurger National Park	1050	[2]	0.58	0.67	1.15	0.57
Indian rhinoceros	Mammalia	Perissodactyla	*Rhinoceros*	*unicornis*	https://www.youtube.com/watch?v=zIgPnuStAuU	Walking Rhino	1900	[11]	0.54	0.74	1.37	0.49
White rhinoceros	Mammalia	Perissodactyla	*Ceratotherium*	*simum*	https://www.youtube.com/watch?v=KBn4BbqrpBQ	Waterberg/Namibia, white rhino walking towards our car	1700	[2]	0.64	0.69	1.09	0.49
White rhinoceros	Mammalia	Perissodactyla	*Ceratotherium*	*simum*	https://www.youtube.com/watch?v=o1zfWmKOzFY	The White Rhino walking past	1700	[2]	0.54	0.72	1.35	0.52
White rhinoceros	Mammalia	Perissodactyla	*Ceratotherium*	*simum*	https://www.youtube.com/watch?v=lyRm3bg7z0E	White Rhino walk past , Lake nakuru , Kenya June 2009	1700	[2]	0.67	0.71	1.05	0.43
Red fox	Mammalia	Carnivora	*Vulpes*	*vulpes*	https://www.youtube.com/watch?v=aI5RuRvRlY0	Young summer fox walking The Sound	6	[2]	1.21	0.63	0.52	0.31
Dingo	Mammalia	Carnivora	*Canis*	*lupus dingo*	https://www.youtube.com/watch?v=2NI4DVblBuc	wild dingo says hello !	16.2	[22]	1.12	0.64	0.57	0.32
Black wolf	Mammalia	Carnivora	*Canis*	*lupus*	https://www.youtube.com/watch?v=tjajo3xnIiM	big wolf walking down highway	40	[13]	0.98	0.68	0.70	0.32

African wild dog	Mammalia	Carnivora	*Lycaon*	*pictus*	https://www.youtube.com/watch?v=ScockAHNnxA	African Wild Dogs female walking	20	[13]	1.01	0.68	0.67	0.32
African wild dog	Mammalia	Carnivora	*Lycaon*	*pictus*	https://www.youtube.com/watch?v=Ku9yMdaZaO0	African Wild Dogs	20	[13]	0.88	0.64	0.73	0.40
African wild dog	Mammalia	Carnivora	*Lycaon*	*pictus*	https://www.youtube.com/watch?v=Ku9yMdaZaO0	African Wild Dogs	20	[13]	0.88	0.64	0.73	0.40
Golden jackal	Mammalia	Carnivora	*Canis*	*aureus*	https://www.youtube.com/watch?v=_k_MJt2HDCY	Jackal walking through hundreds of Waterfowl	10	[13]	0.95	0.66	0.70	0.36
Golden jackal	Mammalia	Carnivora	*Canis*	*aureus*	https://www.youtube.com/watch?v=JMaGL_YeLCc	Golden Jackal (Canis aureus) spotted in Bandhavgarh	10	[13]	1.33	0.61	0.46	0.30
Arctic wolf	Mammalia	Carnivora	*Canis*	*lupus arctos*	https://www.youtube.com/watch?v=XvBgmvLi3E8&list=UUcfQVMHFyx1Ziy1ks6pCRZQ	Arctic Wolves Walking (Slow Motion Animation Reference)	43.5	[17]	0.89	0.69	0.78	0.35
Bat eared fox	Mammalia	Carnivora	*Otocyon*	*megalotis*	https://www.youtube.com/watch?v=TffuPXlCmkQ	Bat Eared Foxes in the Maasai Mara	4.15	[2]	1.16	0.61	0.52	0.34
Crab eating fox	Mammalia	Carnivora	*Cerdocyon*	*thous*	https://www.youtube.com/watch?v=DCQn5VIR3Zc	Crab-eating Fox	6.5	[11]	1.46	0.61	0.42	0.27
Racoon	Mammalia	Carnivora	*Procyon*	*lotor*	https://www.	Just a random	13.5	[23]	1.34	0.63	0.47	0.27

					youtube.com /watch?v=5etVOnQnllM	raccoon walking						
White nosed coati	Mammalia	Carnivora	*Nasua*	*narica*	https://www.youtube.com/watch?v=wHjkcEhujBM	White-Nosed Coati	4.5	[24]	1.57	0.62	0.40	0.24
Racoon	Mammalia	Carnivora	*Procyon*	*lotor*	https://www.youtube.com/watch?v=uohw49UDu_4	Raccoon Walking & Climbing (Slow Motion Animation Reference)	13.5	[23]	0.90	0.70	0.78	0.33
striped skunk	Mammalia	Carnivora	*Maphitis*	*mephitis*	https://www.youtube.com/watch?v=nJTnpE9Tdq8	skunk walk	3	[13]	1.67	0.64	0.39	0.21
Emu	Aves	Casuariiformes	*Dromaius*	*novaehollandiae*	https://www.youtube.com/watch?v=n65l42R6BnM	wild emus walking up the road	34.2	[25]	0.59	0.73	1.25	0.45
Emu	Aves	Casuariiformes	*Dromaius*	*novaehollandiae*	https://www.youtube.com/watch?v=n65l42R6BnM	wild emus walking up the road	34.2	[25]	0.48	0.75	1.57	0.51
Cassowary	Aves	Casuariiformes	*Casuarius*	*casuarius*	https://www.youtube.com/watch?v=01XNqQLLCg4	Southern Cassowaries (*Casuarius casuarius*) at Etty Bay, Queensland, Australia	44	[25]	0.61	0.72	1.18	0.46
Cassowary	Aves	Casuariiformes	*Casuarius*	*casuarius*	https://www.youtube.com/watch?v=hS90OchGPKM	cassowary	44	[25]	0.47	0.76	1.61	0.51
Cassowary	Aves	Casuariiformes	*Casuarius*	*casuarius*	https://www.youtube.com/watch?v=8js	Face to face with a wild Cassowary	44	[25]	0.57	0.72	1.27	0.49

					nDbSXXT8							
Kiwi	Aves	Apterygiformes	*Apteryx*	*australis*	https://www.youtube.com/watch?v=QO7vNzTSZhU	kiwi bird on stewart island	2.33	[25]	0.91	0.64	0.70	0.40
Kiwi	Aves	Apterygiformes	*Apteryx*	*australis*	https://www.youtube.com/watch?v=lo-01mgVyBM	Kiwis@stewart island.m4v	2.33	[25]	1.16	0.66	0.57	0.29
Rhea	Aves	Rheiformes	*Rhea*	*americana*	https://www.youtube.com/watch?v=-X7zyymfRDM	rhea walk	23	[25]	0.63	0.72	1.14	0.44
Ostrich	Aves	Struthioniformes	*Struthio*	*camelus*	https://www.youtube.com/watch?v=MuCDACxKj3I	131110 2 Sacramento Zoo an ostrich walking	111	[25]	0.43	0.81	1.87	0.43
Lesser rhea	Aves	Rheiformes	*Rhea*	*pennata*	https://www.youtube.com/watch?v=PhJrmMz9wQQ	Lesser or Puna Rhea (*Rhea pennata*)	23.9	[25]	0.64	0.71	1.12	0.45
Peacock	Aves	Galliformes	*Pavo*	*cristatus*	https://www.youtube.com/watch?v=mJ1dJyzVi1c	peacock walking freely - peacock bird	4.76	[25]	0.69	0.69	1.00	0.44
Peacock	Aves	Galliformes	*Pavo*	*cristatus*	https://www.youtube.com/watch?v=DpyJxma0kig	peacock walking around	4.76	[25]	1.05	0.67	0.64	0.31
Peacock	Aves	Galliformes	*Pavo*	*cristatus*	https://www.youtube.com/watch?v=3yo9lV_zqWE	peacocks running, flying and dancing in my garden	4.76	[25]	0.83	0.74	0.90	0.31
Peacock	Aves	Galliformes	*Pavo*	*cristatus*	https://www.youtube.com	peacocks running, flying and dancing in	4.76	[25]	1.03	0.67	0.66	0.32

					/watch?v=3yo9lV_zqWE	my garden					
Helmeted Guinea fowl	Aves	Galliformes	*Numida*	*meleagris*	https://www.youtube.com/watch?v=qw1UUbEd9nM	guinea fowl	1.299	[25]	0.94	0.68	0.73	0.34
Helmeted Guinea fowl	Aves	Galliformes	*Numida*	*meleagris*	https://www.youtube.com/watch?v=qw1UUbEd9nM	guinea fowl	1.299	[25]	1.17	0.69	0.59	0.26
Common pheasant	Aves	Galliformes	*Phasianus*	*colchicus*	https://www.youtube.com/watch?v=QWiMKbNaCFo	pheasant confused by parked car	1.317	[25]	0.79	0.71	0.89	0.37
Turkey	Aves	Galliformes	*Meleagris*	*gallopavo*	https://www.youtube.com/watch?v=gp_c-b-pSkI	turkey walking may 11 2011	7.8	[25]	0.46	0.73	1.58	0.58
Turkey	Aves	Galliformes	*Meleagris*	*gallopavo*	https://www.youtube.com/watch?v=qvDPldVugKA	turkeys walking in the road	7.8	[25]	0.82	0.75	0.91	0.31
Turkey	Aves	Galliformes	*Meleagris*	*gallopavo*	https://www.youtube.com/watch?v=qcBLvM_ty1E	Wild turkey walking down the street in Akron, Ohio	4.3	[25]	0.91	0.69	0.76	0.34
Australian brush turkey	Aves	Galliformes	*Alectura*	*lathami*	https://www.youtube.com/watch?v=Xr3lEVPl0wY	Beautiful Australian Bush Turkey - A chance encounter!	2.52	[25]	1.00	0.67	0.67	0.32
Chukar partridge	Aves	Galliformes	*Alectoris*	*chukar*	https://www.youtube.com/watch?v=4T_VJNx7WW4	The chukar partridge (*Alectoris chukar*)	4.66	[25]	0.99	0.62	0.63	0.38
Chukar	Aves	Galliforme	*Alectoris*	*chukar*	https://www.	chukar partridge	4.66	[25]	0.88	0.66	0.75	0.39

partridge		s			youtube.com/watch?v=K9G50lOtoK0							
Chukar partridge	Aves	Galliformes	*Alectoris*	*chukar*	https://www.youtube.com/watch?v=-FZdV7BJzaQ	Chukar partridge in the yard (includes their call)	4.66	[25]	1.22	0.64	0.53	0.29
Red junglefowl	Aves	Galliformes	*Gallus*	*gallus*	https://www.youtube.com/watch?v=y1lRTFAZiQw	Kong's Southeast Asian Pure Red Junglefowls	0.99	[25]	0.77	0.62	0.81	0.49
Sri Lankan junglewfowl	Aves	Galliformes	*Gallus*	*lafayettii*	https://www.youtube.com/watch?v=3B10h2lPzFM	sri lanka junglefowl	0.97	[25]	1.35	0.63	0.47	0.27
Bare-faced curassow	Aves	Galliformes	*Crax*	*fasciolata*	https://www.youtube.com/watch?v=jdO0f_MkoQY	Casal de mutum, Crax fasciolata, Bare-faced Curassow, Mutum de penacho,	2.6	[25]	0.84	0.71	0.84	0.35
Bare-faced curassow	Aves	Galliformes	*Crax*	*fasciolata*	https://www.youtube.com/watch?v=laR6kEanc60	Bare-faced Curassow, Crax fasciolata, Brazilian fauna, fauna,	2.6	[25]	0.65	0.66	1.02	0.52
Bare-faced curassow	Aves	Galliformes	*Crax*	*fasciolata*	https://www.youtube.com/watch?v=NUD4wtGjzmM	Couple curassows, Bare-faced Curassow, Crax fasciolata, Fauna,	2.6	[25]	1.21	0.74	0.61	0.21
Great curassow	Aves	Galliformes	*Crax*	*rubra*	https://www.youtube.com/watch?v=KKG2uT-jEWo	Bosque del Cabo, Costa Rica - Birds	4.13	[25]	0.92	0.70	0.76	0.33
Great curassow	Aves	Galliformes	*Crax*	*rubra*	https://www.youtube.com/watch?v=XzOiEZf_Qag	Great Curassow (*Crax rubra*)	4.13	[25]	0.63	0.78	1.24	0.34

Helmeted curassow	Aves	Galliformes	*Pauxi*	*pauxi*	https://www.youtube.com/watch?v=g8fxcL2XW7A	northern helmet curassow in barred phase	2.7	[25]	0.77	0.69	0.89	0.40
Australian brush turkey	Aves	Galliformes	*Alectura*	*lathami*	https://www.youtube.com/watch?v=llA_F4GwVmo	Camping at Deep Water National Park	2.52	[25]	0.97	0.66	0.67	0.35
Silver pheasant	Aves	Galliformes	*Lophura*	*nycthemera*	https://www.youtube.com/watch?v=2waNeMsqtXk	Silver Pheasant	1.75	[25]	0.75	0.68	0.90	0.43
Silver pheasant	Aves	Galliformes	*Lophura*	*nycthemera*	https://www.youtube.com/watch?v=5y_ss0yf41g	silver pheasant display	1.75	[25]	0.85	0.63	0.73	0.44
Golden pheasant	Aves	Galliformes	*Chrysolophus*	*pictus*	https://www.youtube.com/watch?v=M9Mn8ESljoo	Red Golden Pheasant - Golden Pheasants or Chinese Pheasants in Islamabad Zoo	0.64	[25]	0.83	0.66	0.79	0.41
Golden pheasant	Aves	Galliformes	*Chrysolophus*	*pictus*	https://www.youtube.com/watch?v=cOSoLJwvGCI	Golden Pheasant and another bird	0.64	[25]	0.81	0.72	0.89	0.34
Copper pheasant	Aves	Galliformes	*Syrmaticus*	*soemmerringii*	https://www.youtube.com/watch?v=bGEXPif66cA	copper pheasant (*syrmaticus soemmerringii ijimae*)	0.9	[25]	1.43	0.66	0.46	0.24
Copper pheasant	Aves	Galliformes	*Syrmaticus*	*soemmerringii*	https://www.youtube.com/watch?v=bGEXPif66cA	copper pheasant (*syrmaticus soemmerringii ijimae*)	1.3	[25]	1.26	0.61	0.48	0.31
Willow ptarmigan	Aves	Galliformes	*Lagopus*	*lagopus*	https://www.youtube.com/watch?v=gcBEahZhyxU	Willow Ptarmigan in Denali Park (Alaska)	0.61	[25]	1.63	0.69	0.42	0.19

Canada goose	Aves	Anseriformes	*Branta*	*canadensis*	https://www.youtube.com/watch?v=zg1g4zi7Ktg	Canadian Geese Walk With Goslings (Slow Motion Animation Reference)	3.81	[25]	1.08	0.76	0.70	0.23
Bar-headed goose	Aves	Anseriformes	*Anser*	*indicus*	https://www.youtube.com/watch?v=wEysp9v75ho	Bar-headed Goose - one of the world's highest-flying birds	2.46	[25]	0.66	0.73	1.10	0.41
Barnacle goose	Aves	Anseriformes	*Branta*	*leucopsis*	https://www.youtube.com/watch?v=GDYof2VjJWg	barnacle geese - the everyday life	1.79	[25]	0.94	0.67	0.71	0.35
Barnacle goose	Aves	Anseriformes	*Branta*	*leucopsis*	https://www.youtube.com/watch?v=GDYof2VjJWg	barnacle geese - the everyday life	1.79	[25]	1.01	0.73	0.72	0.27
Barnacle goose	Aves	Anseriformes	*Branta*	*leucopsis*	https://www.youtube.com/watch?v=GDYof2VjJWg	barnacle geese - the everyday life	1.79	[25]	1.23	0.73	0.59	0.22
Barnacle goose	Aves	Anseriformes	*Branta*	*leucopsis*	https://www.youtube.com/watch?v=yXedtMyE1EA	Barnacle geese on Solway Firth estuary Cumbria	1.79	[25]	0.79	0.73	0.92	0.34
Mute swan	Aves	Anseriformes	*Cygnus*	*olor*	https://www.youtube.com/watch?v=kF5gdWGUekA	walking swan	11.8	[25]	0.62	0.81	1.31	0.31
Mallard	Aves	Anseriformes	*Anas*	*platyrhyncos*	https://www.youtube.com/watch?v=uRTi4fy1od0	mallard duck walking on beach quacking	1.25	[25]	1.76	0.66	0.38	0.19
Mallard	Aves	Anseriformes	*Anas*	*platyrhyncos*	https://www.youtube.com/watch?v=ZJMfazYPwpY	marvellous mallard mates relaxing and walking together	1.25	[25]	1.31	0.66	0.50	0.26
Mallard	Aves	Anserifor	*Anas*	*platyrhync*	https://www.	walking ducklings	1.1	[25]	2.10	0.65	0.31	0.17

		mes		*os*	youtube.com /watch?v=jwHAHqlLgyw							
Mallard	Aves	Anseriformes	*Anas*	*platyrhyncos*	https://www.youtube.com/watch?v=GjuB0Kmk_BA	ducks walking in the street	1.1	[25]	2.03	0.64	0.31	0.18
Pekin duck	Aves	Anseriformes	*Anas*	*platyrhyncos domestica*	https://www.youtube.com/watch?v=XZVF0VS0VNQ	peking ducks walking	4.3	[25]	1.06	0.73	0.69	0.25
Pekin duck	Aves	Anseriformes	*Anas*	*platyrhyncos domestica*	https://www.youtube.com/watch?v=XZVF0VS0VNQ	peking ducks walking	4.3	[25]	1.44	0.76	0.53	0.17
Pekin duck	Aves	Anseriformes	*Anas*	*platyrhyncos domestica*	https://www.youtube.com/watch?v=g139Ud9B1Pg	pekin duck	4.3	[25]	1.58	0.73	0.46	0.17
Pekin duck	Aves	Anseriformes	*Anas*	*platyrhyncos domestica*	https://www.youtube.com/watch?v=QX04X0nrF1A	stanley the male pekin duck	4.3	[25]	1.35	0.79	0.59	0.15
Black swan	Aves	Anseriformes	*Cygnus*	*atratus*	https://www.youtube.com/watch?v=HTPL8HHsT_0	Pekin Ducks & Black Swans in Rancho Mirage Lake, Ca.	6.2	[25]	0.61	0.80	1.32	0.33
Black swan	Aves	Anseriformes	*Cygnus*	*atratus*	https://www.youtube.com/watch?v=HTPL8HHsT_0	Pekin Ducks & Black Swans in Rancho Mirage Lake, Ca.	6.2	[25]	0.55	0.84	1.54	0.29
Emperor penguin	Aves	Sphenisciforme	*Aptenodytes*	*forsteri*	https://www.youtube.com/watch?v=tF8Jg1zIG2E	emperor penguin sf2199	38.2	[25]	1.01	0.83	0.82	0.17
Emperor penguin	Aves	Sphenisciforme	*Aptenodytes*	*forsteri*	https://www.youtube.com	Penguin Walking on Ice l Penguin in	38.2	[25]	1.31	0.68	0.52	0.24

					/watch?v=UEET_t1IbLM	Antarctica l Penguin Noises l Fat Penguin Walking						
Gentoo penguin	Aves	Sphenisciforme	*Pygoscelis*	*papua*	https://www.youtube.com/watch?v=z6dCxE7_5p0	Gentoo penguin walking at Neko Harbor on the Antarctic Peninsula	6.4	[25]	1.46	0.69	0.47	0.21
Gentoo penguin	Aves	Sphenisciforme	*Pygoscelis*	*papua*	https://www.youtube.com/watch?v=lk57Blifn6s	Gentoo Penguin Walking on Brown Bluff, Antarctica	6.4	[25]	1.88	0.59	0.31	0.22
Gentoo penguin	Aves	Sphenisciforme	*Pygoscelis*	*papua*	https://www.youtube.com/watch?v=l3i6z5iFMcQ	gentoo penguins	6.4	[25]	1.66	0.63	0.38	0.22
Chinstrap penguin	Aves	Sphenisciforme	*Pygoscelis*	*antarctica*	https://www.youtube.com/watch?v=XfsV-kQJYEo	The Chinstrap Penguin Walk	4.4	[25]	1.68	0.65	0.39	0.21
Chinstrap penguin	Aves	Sphenisciforme	*Pygoscelis*	*antarctica*	https://www.youtube.com/watch?v=XfsV-kQJYEo	The Chinstrap Penguin Walk	4.4	[25]	2.00	0.62	0.31	0.19
Chinstrap penguin	Aves	Sphenisciforme	*Pygoscelis*	*antarctica*	https://www.youtube.com/watch?v=XfsV-kQJYEo	The Chinstrap Penguin Walk	4.4	[25]	1.58	0.72	0.46	0.18
Chinstrap penguin	Aves	Sphenisciforme	*Pygoscelis*	*antarctica*	https://www.youtube.com/watch?v=Q_bKCnU-NPg	Chinstrap penguin walking 2	4.4	[25]	1.42	0.68	0.48	0.23
King penguin	Aves	Sphenisciforme	*Aptenodytes*	*patagonicus*	https://www.youtube.com/watch?v=3gK9pyewiYQ	king penguins walking	12.4	[25]	1.38	0.68	0.49	0.23
King penguin	Aves	Sphenisciforme	*Aptenodytes*	*patagonicus*	https://www.youtube.com	king penguins walking	12.4	[25]	1.10	0.67	0.61	0.30

					/watch?v=3g K9pyewiYQ							

Supplementary Table S2: Slopes and intercepts of the ANCOVA analysis (of the form $Y = aX^b$, where a is the intercept and b is the slope) describing the variation in kinematic parameters accounted for by class group and body mass (M_b, kg) for avian and mammalian classes: duty factor (DF), stride frequency (f_{stride}, Hz), stance duration (t_{stance}, s) and swing duration (t_{swing}, s). Numbers in bold indicate values which are significantly different.

			Full model								Minimum adequate model							
Parameter	Covariate	Group	Intercept	SE intercept	T intercept	p intercept	Slope	SE slope	T slope	p slope	Intercept	SE intercept	T intercept	p intercept	Slope	SE slope	T slope	p slope
ln(DF)	ln(M_b)	**mammal**	**0.62**	0.017	-57.3	4.8×10^{-6}	**0.02**	0.006	4.9	0.020	-	-	-	-	-	-	-	-
		**bird**	**0.67**	0.017	-47.9	4.8×10^{-6}	**0.03**	0.006	9.4	0.020	-	-	-	-	-	-	-	-
ln(f_{stride})	ln(M_b)	**mammal**	1.62	0.074	13.1	8.3×10^{-5}	-0.15	0.028	-10.9	0.89	**1.62**	0.061	15.2	1.9×10^{-8}	-0.15	0.010	-15.1	4.9×10^{-36}
		**bird**	1.21	0.074	5.1	8.3×10^{-5}	-0.15	0.028	-10.6	0.89	**1.21**	0.061	3.5	1.9×10^{-8}	-0.15	0.010	-15.1	4.9×10^{-36}
ln(t_{stance})	ln(M_b)	**mammal**	0.38	0.082	-23.6	7.8×10^{-6}	0.17	0.031	10.8	0.74	**0.38**	0.068	-26.6	8.3×10^{-12}	0.17	0.011	15.2	2.4×10^{-36}
		**bird**	0.55	0.082	-14.5	7.8×10^{-6}	0.18	0.031	11.5	0.74	**0.56**	0.068	-12.1	8.3×10^{-12}	0.17	0.011	15.2	2.4×10^{-36}
ln(t_{swing})	ln(M_b)	**mammal**	0.24	0.069	-41.8	0.04	0.12	0.026	9.3	0.05	0.25	0.057	-41.5	0.38	0.11	0.009	12.0	5.5×10^{-26}
		**bird**	0.27	0.069	-37.7	0.04	0.07	0.026	5.4	0.05	0.26	0.057	-39.7	0.38	0.11	0.009	12.0	5.5×10^{-26}

Supplementary Table S3: Trackway data for walking bipedal and quadrupedal dinosaurs used in the calculation of Froude number. Only Froude numbers of ≤ 0.5 were included as above these values animals are likely to be running. Values for ornithopods in the cited literature were not included.

Speed (m/s)	Hip height (m)	Froude number	Category	Reference
2	2.1	0.19	biped	[26]
2.2	2	0.25	biped	[26]
1.2	1.1	0.13	biped	[26]
2.2	1.25	0.39	biped	[26]
1.3	0.97	0.18	biped	[27]
1.8	0.93	0.36	biped	[27]
1.5	0.9	0.25	biped	[27]
1.7	1.01	0.29	biped	[27]
1.5	1.04	0.22	biped	[27]
1.4	2.38	0.08	biped	[27]
1.3	1.73	0.10	biped	[27]
2	2.6	0.16	biped	[28]
2.3	2.6	0.21	biped	[29]
2.05	1.8	0.24	biped	[29]
2.4	1.5	0.39	biped	[29]
0.7	1.7	0.03	biped	[29]
2.2	1.8	0.27	biped	[29]
2.5	2.2	0.29	biped	[29]
0.9	2.3	0.04	biped	[29]
1.9	2.6	0.14	biped	[29]
2.7	3.6	0.21	biped	[29]
0.8	0.17	0.38	biped	[29]
0.9	0.2	0.41	biped	[29]
0.5	0.69	0.04	biped	[29]
1.6	0.82	0.32	biped	[29]
2.1	1.07	0.42	biped	[29]
1.3	1.37	0.13	biped	[29]
1.3	1.56	0.11	biped	[29]
1.25	0.64	0.25	biped	[30]
2.6	1.90	0.36	biped	[31]
2.5	1.76	0.36	biped	[31]
2.5	1.67	0.38	biped	[31]
2.5	1.69	0.38	biped	[31]
2.6	1.50	0.46	biped	[31]
2.5	1.45	0.44	biped	[31]
1.8	1.43	0.23	biped	[31]
1	3	0.03	quadruped	[26]
1.1	1.5	0.08	quadruped	[26]
0.6	3.67	0.01	quadruped	[27]
0.5	2.64	0.01	quadruped	[27]
0.9	1.4	0.06	quadruped	[29]

Supplementary Information References

1 Day, L. M. & Jayne, B. C. Interspecific scaling of the morphology and posture of the limbs during the locomotion of cats (Felidae). *J. Exp. Biol.* **210**, 642-654, doi:0.1242/Jeb.02703 (2007).

2 Kingdon, J. *The Kingdon field guide to African mammals*. (Bloomsbury Publishing Plc, 2012).

3 Smith, R. J. & Jungers, W. L. Body mass in comparative primatology. *J. Hum. Evol.* **32**, 523-559, doi:10.1006/Jhev.1996.0122 (1997).

4 Alexander, R. M. & Maloiy, G. M. O. Stride lengths and stride frequencies of primates. *J. Zool.* **202**, 577-582 (1984).

5 Stirling, I., Spencer, C. & Andriashek, D. Immobilization of polar bears (*Ursus Maritimus*) with telazol in the Canadian Arctic. *J. Wildl. Dis.* **25**, 159-168 (1989).

6 Blanchard, B. M. in *Int. Conf. Bear Res. and Manage.* 99-107.

7 Pelton, M. R. in *Wild mammals of North America: Biology, management and conservation* (eds J.A. Chapman & G.A. Feldhamer) 547-555 (JHU Press, 2003).

8 Yerena, E. & Torres, D. in *Int. Conf. Bear Res. and Manage.* 169-172.

9 Wong, S. T., Servheen, C. W. & Ambu, L. Home range, movement and activity patterns, and bedding sites of Malayan sun bears *Helarctos malayanus* in the Rainforest of Borneo. *Biol. Conserv.* **119**, 169-181, doi:10.1016/j.biocon.2003.10.029 (2004).

10 Johnson, K. G., Schaller, G. B. & Hu, J. C. Comparative behavior of red and giant pandas in the Wolong reserve, China. *J. Mammal.* **69**, 552-564, doi:10.2307/1381347 (1988).

11 Nowak, R. M. *Walker's mammals of the world*. 5 edn, (JHU Press, 1991).

12 Kurtu, M. Y. An assessment of the productivity for meat and the carcass yield of camels (*Camelus dromedarius*) and of the consumption of camel meat in the eastern region of Ethiopia. *Trop. Anim. Health Prod.* **36**, 65-76, doi:10.1023/B:Trop.0000009520.34657.35 (2004).

13 Garland, T. The relation between maximal running speed and body mass in terrestrial mammals. *J. Zool.* **199**, 157-170 (1983).

14 Massei, G., Genov, P. V. & Staines, B. W. Diet, food availability and reproduction of wild boar in a Mediterranean coastal area. *Acta Theriol. (Warsz).* **41**, 307-320 (1996).

15 Woods, C. A. *Erethizon dorsatum*. 1-6 (The American society of mammologists, 1973).

16 Mones, A. & Ojasti, J. *Hydrochoerus hydrochaeris*. 1-7 (The American society of mammologists, 1986).

17 Macdonald, D. W. *The encyclopedia of mammals*. (Oxford University Press, 2009).

18 Clauss, M. *et al.* The maximum attainable body size of herbivorous mammals: morphophysiological constraints on foregut, and adaptations of hindgut fermenters. *Oecologia* **136**, 14-27,

doi:10.1007/s00442-003-1254-z (2003).
19 Hansen, R. M., Mugambi, M. M. & Bauni, S. M. Diets and trophic ranking of ungulates of the northern Serengeti. *J. Wildl. Manage.* **49**, 823-829, doi:10.2307/3801717 (1985).
20 Luxenberg, S. *Tapirus terrestris* (2014) Available at: <http://animaldiversity.ummz.umich.edu/accounts/Tapirus_terrestris/> (Accessed: August 18th 2014).
21 Khan, M. M. *The Malayan tapir*. 4 (Institut Terjemahan and Buku Malaysia Berhad, 1935).
22 Green, B. Estimation of food consumption in dingo, *Canis familiaris dingo*, by means of ^{22}Na turnover. *Ecology* **59**, 207-210, doi:10.2307/1936363 (1978).
23 Whitaker, J. O. *National Audubon Society field guide to North American mammals* 2edn, 942 (Alfred A. Knopf, Inc, 1996).
24 Schmidly, D. J. *The mammals of Texas*. (University of Texas Press, 2004).
25 Dunning, J. B. *CRC handbook of avian body masses*. 2 edn, (CRC press, 2008).
26 Alexander, R. M. Estimates of speeds of dinosaurs. *Nature* **261**, 129-130 (1976).
27 Mazzetta, G. V. & Blanco, R. E. Speeds of dinosaurs from the Albian-Cenomanian of Patagonia and sauropod stance and gait. *Acta Palaeontol. Pol.* **46**, 235-246 (2001).
28 Alexander, R. M. *Dynamics of dinosaurs and other extinct giants*. (Columbia University Press, 1989).
29 Thulborn, R. A. & Wade, M. Dinosaur trackways in the Winton formation (Mid-Cretaceous) of Queensland. *Mem. Queensl. Mus.* **21**, 413-517 (1984).
30 Tucker, M. E. & Burchette, T. P. Triassic dinosaur footprints from South Wales: Their context and preservation. *Palaeogeogr., Palaeoclimatol., Palaeoecol.* **22**, 195-208, doi:10.1016/0031-0182(77)90028-1 (1977).
31 Farlow, J. O. Estimates of dinosaur speeds from a new trackway site in Texas. *Nature* **294**, 746-748 (1981).
